# Community-led standards for global wastewater-based infectious disease surveillance

**DOI:** 10.1371/journal.pgph.0006266

**Published:** 2026-04-20

**Authors:** Emily A. Smith, Maryam Ahmadi Jeshvaghane, Dylan A. Pilz, Mercury Shitindo, João Costa da Silva, Lance Gable, Keaghan Brown, Tracey Calvert-Joshua, Farzaana Diedericks, Gültekin Ünal, Jillian S. Paull, Nitin B. Shukla, Zeenatuddeen Muhammad, Justice Ohene Amofa, Elyssa Jiawen Liu, Emma J. Griffiths, Chrystal Landgraff, Fatma Z. Guerfali, Nicki Tiffin, Joshua I. Levy, on behalf of the Public Health Alliance for Genomic Epidemiology (PHA4GE) Wastewater Surveillance Working Group

**Affiliations:** 1 Center for Infectious Disease Research and Policy, University of Minnesota, Minneapolis, Minnesota, United States of America; 2 Department of Translational Medicine, The Scripps Research Institute, La Jolla, California, United States of America; 3 Africa Bioethics Network, Nairobi, Kenya; 4 Faculty of Health Sciences, University of Zaragoza, Zaragoza, Spain; 5 Public Health Alliance for Genomic Epidemiology, South African National Bioinformatics Institute, University of the Western Cape, Cape Town, South Africa; 6 Wayne State University Law School, Detroit, Michigan, United States of America; 7 Department of Microbiology, Faculty of Veterinary Medicine, Ankara University, Ankara, Turkey; 8 Howard Hughes Medical Institute, Chevy Chase, Maryland, United States of America; 9 Broad Institute of MIT and Harvard, Cambridge, Massachusetts, United States of America; 10 Gujarat Biotechnology Research Centre, Gandhinagar, Gujarat, India; 11 Environmental Health Council of Nigeria, Abuja, Nigeria; 12 Genomics and Bioinformatics Core Facility Unit, Noguchi Memorial Institute for Medical Research, University of Ghana, Accra, Ghana; 13 Centre for Outbreak Preparedness, Duke-NUS Medical School, Singapore, Singapore; 14 Centre for Infectious Disease Genomics and One Health, Faculty of Health Sciences, Simon Fraser University, Vancouver, British Columbia, Canada; 15 National Microbiology Laboratory, Public Health Agency of Canada, Winnipeg, Manitoba, Canada; 16 Laboratory of Transmission, Control and Immunobiology of Infections, Institut Pasteur de Tunis, Université de Tunis El Manar, Tunis, Tunisia; 17 South African National Bioinformatics Institute, University of the Western Cape, Cape Town, South Africa; Child Health Research Foundation, BANGLADESH

## Abstract

The COVID-19 pandemic accelerated global adoption of wastewater and environmental surveillance (WES) and revealed its value as a complement to case-based infectious disease monitoring. However, practical scientific, ethical, and legal standards for public health implementation remain limited. To address this gap, the Public Health Alliance for Genomic Epidemiology (PHA4GE) established a multidisciplinary international working group to develop community-driven, open guidance on WES methods and best practices. By integrating expertise from public health researchers, practitioners, bioethicists, and legal scholars, together with feedback from the broader public health community, these standards aim to promote transparent, reproducible, and accessible implementation of WES worldwide. This continuously updated framework spans surveillance strategies, data analysis, data sharing, and ethical-legal considerations, aligning with normative guidance from global public health authorities while remaining adaptable to diverse contexts and resource levels. All guidance documents can be found at https://pha4ge.github.io/wastewater-guidance.

## Introduction

Wastewater and environmental surveillance (WES) has emerged as a powerful complement to traditional public health monitoring, providing low-cost, population-level insights into disease burden [1–3]. Global adoption of WES accelerated rapidly over the COVID-19 pandemic, spanning diverse deployment contexts and pathogen targets [4–7].

This rapid uptake has led to a proliferation of wastewater methods and tools, but practical and ethical implementation standards are still in the process of being defined [8, 9]. Ongoing efforts including by the World Health Organization (WHO) and the Global Consortium for Wastewater and Environmental Surveillance for Public Health (GLOWACON) have begun to establish normative guidelines, however, these emerging standards lack detailed, context-specific guidance needed for implementation, particularly in resource-limited settings [10–12]. Development of guidance that captures the diverse scenarios in which wastewater surveillance is deployed requires the public health community, including scientists and stakeholders worldwide, to be open to public discussion and continuous, transparent refinement. Therefore, community-led, participatory frameworks are essential to ethical and effective implementation worldwide [13].

**Fig 1:**
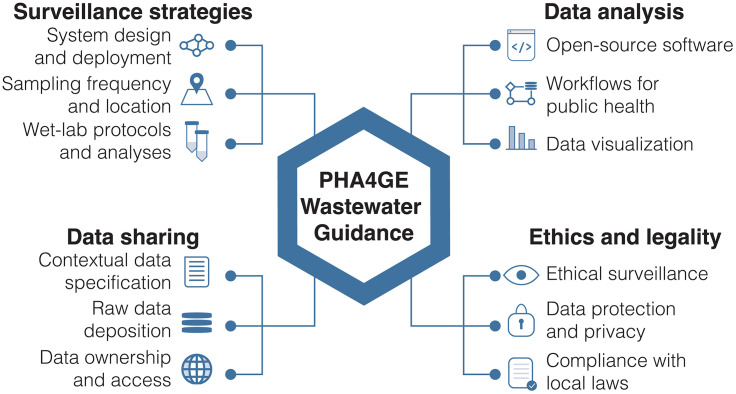
Overview of PHA4GE WES guidance and standards development The PHA4GE wastewater guidance document is organized into four main sections. The surveillance strategies section describes standardized protocols for WES design and implementation, including sampling frequency, site selection, and laboratory processing. The data sharing section outlines best practices for raw data management, ownership and access, and effective dissemination. The data analysis section summarizes tiered workflows, open-source tools, and visualization approaches to support broadly interpretable results. The ethics and legality section addresses key considerations for equitable, local community-engaged surveillance, including protection of individual privacy and alignment with local regulations.

To ensure WES is sustainably and equitably integrated into public health systems, standards need to be actionable, adaptable, and informed by practitioners from a wide range of implementation contexts, providing corresponding validated workflows that minimize dependence on local technical expertise to achieve high-quality, interpretable outputs. To address this need, we organized an interdisciplinary, community-led working group within the Public Health Alliance for Genomic Epidemiology (PHA4GE) composed of epidemiologists, laboratory technicians, bioinformaticians, bioethicists, public health officials, and legal experts, dedicated to providing public-facing, community-led, live guidance documents on wastewater surveillance tools, protocols, and best practices (Fig 1) [14]. During biweekly meetings, working group members discuss key challenges and outstanding questions, set priorities based on group experience and external feedback, and vote on proposed updates to the guidance documents.

Here, we describe the objectives and progress of our working group in developing practical, open guidance for wastewater surveillance, including for data analysis, data sharing, and ethical implementation, and discuss outstanding challenges for global public health use. We aim to build on existing guidance, including from WHO and GLOWACON, to provide validated and practitioner-friendly approaches, workflows, and decision tools. By developing open standards and resources in close collaboration with public health community researchers actively applying them, we aim to foster practical, transparent, and equitable approaches to improve infectious disease surveillance worldwide.

## Expanding access to transparent, robust, and reproducible analyses

Historically, leading public health institutions such as US Centers for Disease Control and Prevention (US CDC) have developed “gold-standard” assays and workflows to identify and characterize pathogens, but these tend to be limited in scope (e.g., just for clinical detection) and in effectiveness across contexts. To expand surveillance capabilities beyond these established tools and enable use of a wider range of workflows for monitoring disease spread, including WES, public health labs increasingly rely on open-source resources including laboratory protocols, sequencing approaches, and bioinformatics pipelines. These resources are contributed by labs across academia, industry, and public health, but rarely include formal validation or performance guarantees.

Open-source software forms the foundation of public health bioinformatics, and recent community efforts – mostly volunteer-based – including the State Public Health Bioinformatics Group (STaPH-B) and PHA4GE have emerged with the aim to enable anyone, anywhere to analyze data using the same validated and standardized tools. Similar, though less coordinated efforts have emerged for sample collection and laboratory processing steps, and are accessible through open platforms such as protocols.io [15,16]. Likewise, tools for low-cost sequencing, including open source amplicon sequencing primer schemes, such as those developed by the ARTIC network, have become a core component of both wastewater and clinical sequencing workflows, and are regularly updated and public health community-validated [17].

In our guidance documents, we provide community-vetted resources that can support end-to-end surveillance, from sample collection and analysis to downstream reporting, and that are adaptable to available resources. To ensure equitable access to high-quality bioinformatics pipelines that abide by best practices [18], we include “choose your own adventure”-style recommendations to enable public health scientists, from novices to experts, to perform state-of-the-art analyses using expertise-level tailored tools and workflows. We establish similar public health community-driven approaches for core components of wastewater surveillance, including sampling strategy and data dissemination. Because these resources are fully community-driven and open-source, anyone can propose updates or refinements to the existing guidance documents, ensuring that these resources continue to evolve along with technological advances and changes in public health needs.

## Effective wastewater data sharing for global public health

The value of infectious disease surveillance depends on how effectively data and insights are shared from laboratories to public health authorities, decision-makers, and local community members represented in monitored catchments. When shared promptly and transparently, surveillance data can guide public health responses worldwide, inform policy, and build public trust. This requires clear mechanisms to translate surveillance data into prevention and treatment responses, including rapid deployment of countermeasures, treatment, and non-pharmaceutical interventions [19,20]. This translation is crucial across pathogens, from recent targets such as SARS-CoV-2 to poliovirus, for which WES has been used to guide vaccination efforts for decades [21,22].

## Technical and structural barriers to effective data sharing

Institutional data siloing, unclear data governance, and a lack of appropriate platforms for sharing sensitive and potentially misinterpretable public health data remain major challenges for many surveillance programs. To enable sharing of findings, public health programs have begun to develop dedicated web-based dashboards [23,24] and scalable frameworks for cross-institutional data integration and visualization [25–27]. However, these platforms remain most suited for educated and web-literate audiences, and further adaptation is needed to ensure broad accessibility and interpretability [28]. Additionally, because surveillance data often have implications that extend beyond local or national contexts, and large-scale international sharing of data and findings is needed to inform understanding of global pathogen evolution and spread.

Sharing wastewater sequence data presents additional challenges, both because of sample complexity and the importance of sampling context. Wastewater samples typically contain mixtures of pathogens that can make genome assembly infeasible, requiring sharing of raw sequence data rather than representative genomes [29]. To support sharing of data and “contextual” metadata, PHA4GE and international partners spanning low, medium, and high-resource countries have developed a “Wastewater Contextual Data Specification” for ontology-based, standardized terminology, and tools and templates to guide submission to public repositories [30]. The specification captures essential information on sampling location, collection and processing methods, and sequencing approach, all of which are critical for meaningful data interpretation and re-use for public health. By enabling consistent, specimen type-specific metadata, this standard promotes effective data sharing, interoperability, and harmonization, maximizing the utility of non-traditional epidemiological data such as wastewater for public health.

## Toward equitable and sustainable wastewater data sharing

In practice, public health data sharing must balance the benefits of open sharing with the need to minimize real-world risks such as misinterpretation and stigmatization, and is further constrained by institutional and governmental regulations. Restricted-use databases such as Pathoplexus and GISAID have enabled controlled yet broad sharing of genome assembly data; however, no analogous infrastructure currently exists for wastewater surveillance data, for which genome assemblies are usually not appropriate due to intra-species diversity in most samples. Leading sequence databases comprising the International Nucleotide Sequence Data Collaboration (INSDC) including NCBI SRA, ENA, and DDBJ are yet to be adapted for the use of wastewater in public health, and there is no public health community-led standard for how sequencing data should be shared with the public, nor any limitations on how the data may be used. Although NCBI has taken steps to incorporate the PHA4GE wastewater specification for data deposition [31], the only option for raw sequence data sharing via INSDC databases is to do so fully openly. This may be inappropriate in many public health contexts, particularly when data re-use could lead to social or economic harms including through travel bans [32], and may discourage timely sharing as data submitters delay sharing until they have published their analyses [33]. Adapting existing restricted-use sharing frameworks such as Pathoplexus will be key to supporting WES data sharing at the global scale.

As public health data sharing expands, additional tensions emerge between maximizing global access and protecting the rights and benefits afforded to data submitters. General frameworks for equitable data sharing have been proposed [34], and more countries are starting to ratify the Nagoya Protocol, an international agreement protecting the rights of countries over their genetic resources and regulating benefit-sharing, but we do not yet have a sustainable data sharing solution for wastewater data. The absence of harmonized frameworks not only limits data comparability but also entrenches inequities, disadvantaging lower-resourced settings in global pathogen surveillance networks that often do not share in the benefits of data sharing, including access to vaccines developed using their data [35].

## Ethical surveillance requires consideration of public health context

Wastewater surveillance can strengthen evidence-based public health responses and communication, but its ethical use requires careful consideration of deployment strategy, cultural and infrastructural context, and approach for data management and dissemination. For the most part, these considerations largely align with established principles of public health ethics, including beneficence (promoting public well-being), nonmaleficence (minimizing harm), proportionality (balancing risks and benefits), and distributive justice (fair and equitable allocation of resources) [36], but wastewater surveillance poses some unique ethical challenges.

## Risks of stigmatization and misuse

Wastewater enables cost-effective, population-level monitoring that can guide public health interventions without requiring direct individual participation or healthcare system access, but it does carry real risk of misuse and misinterpretation. Although wastewater data are aggregated and typically non-identifiable at the population level, managing proportional communication of findings, particularly for stigmatized diseases, communities, and linked population groups, can be complex and context-specific [37]. For example, Mpox has had increased burden among vulnerable communities including sex workers and men who have sex with men (MSM), and has emerged as a common target of wastewater surveillance [38]. Recent work has enabled detection and characterization of regional outbreaks, with some studies even achieving highly resolved, building-level sampling. However, messaging and reporting on these findings can lead to substantial public harm, as many countries still have discriminatory laws against these vulnerable communities and informal stigmatization can also have profound social consequences. These risks necessitate careful consideration of sampling resolution, reporting aggregation levels, and local community consultation prior to implementation [28]. Surveillance and prevention efforts that incorporate strong peer and local community engagement, particularly for HIV, have consistently demonstrated improved effectiveness and acceptability [39]. To operationalize these principles, we provide best-practice protocols for community engagement and risk assessment in our guidance documents.

## Transparent and community-centered governance

The complex nature of wastewater data, encompassing microbial and potentially human-derived signals, requires transparent policies on data collection, use, and sharing. Operational transparency and local community engagement are essential for population-level wastewater surveillance, especially for indigenous communities with unique cultural sensitivities and sovereignty considerations. Ensuring that sampling preserves individual anonymity, is population-representative, and supports equitable distribution of resources to those affected, is crucial to ethical implementation of public health surveillance overall, and for maintaining community trust [28]. Ethical implementation also necessitates that findings can be effectively interpreted and translated to benefit the entire population, especially vulnerable groups, including those with limited literacy and web access. Furthermore, adopting data custodianship rather than ownership paradigms, and emphasizing collective responsibility through shared governance mechanisms that meaningfully involve local communities, infrastructure partners, and public health institutions from project conceptualization onward, is essential for respectful and ethical implementation.

Wastewater-specific ethical standards that are flexible and adaptable to global use-cases are still in active development, with several recent efforts proposing guidance frameworks [28,34]. These initiatives emerged during the COVID-19 pandemic and focus on topics including community engagement, stigma, privacy, and data stewardship, but primarily reflect the experiences of high-income countries [40–42]. Building on this foundational work, we aim to provide public health community-led guidance that is adaptable and practical for ethical wastewater surveillance across diverse resource-levels and societal contexts. For every stage of wastewater surveillance implementation, from sampling design to dissemination, we aim to support ethical deliberation to maximize societal benefit while preventing harm.

## Navigating the ever-changing regulatory landscape

WES operates within a permissive regulatory environment, facilitated by existing public health authorities rather than specific legislation. In the United States, programs operate under general public health authorities primarily grounded in state-level statutes and regulations, providing operational flexibility. However, most funding for these programs comes from federal agencies such as the CDC and the Environmental Protection Agency (EPA), for which future sustainability is unclear at present. In contrast, the European Union recently adopted the 2024 revised Urban Wastewater Directive, which explicitly mandates surveillance for specific pathogens including SARS-CoV-2, requiring Member State implementation by 2027. Overall, the global regulatory landscape remains fluid, evolving with political priorities, public health needs, and judicial interpretation.

Across jurisdictions, there is a general absence of mandatory reporting requirements for wastewater surveillance data, contrasting sharply with clinical case reporting obligations. Many institutions treat wastewater surveillance strictly as environmental data, separate from the clinical reporting requirements for notifiable infectious diseases. While this distinction reflects practical considerations between population-level versus individual data, it can also constrain integration with clinical surveillance and slow outbreak response.

While privacy considerations inform ethical implementation practices, legal privacy protections are generally inapplicable or unsupported for wastewater data. Aggregated wastewater data typically fails to satisfy definitions for personal information under established data protection frameworks, including GDPR, POPIA and HIPAA. In the United States, courts have consistently held that individuals have no reasonable expectation of privacy in wastewater once it enters public infrastructure (*e.g., Riverdale Mills Corp. v. United States*, 410 F.3d 448, 1st Cir. 2005), though programs often adopt voluntary ethical safeguards such as avoiding sampling from small catchments that could enable re-identification of individuals or stigmatization of affected groups. This legal consensus supports implementation without requiring individual consent, while maintaining appropriate protections proportional to population size and vulnerability. Ethically, this approach is justifiable provided that appropriate protections proportional to population size and vulnerability are maintained.

## Incorporating guidance principles into public health practice

To highlight practical use of our guidelines, we draw from real-world examples from the scientific literature demonstrating how different sampling locations, site types, frequencies, collection methods, and sequencing approaches have been used to monitor and respond to disease burden in low to high resource settings. These case studies reflect the diverse contexts in which wastewater surveillance is deployed including context-specific adaptations to sampling and analysis, and encompass many different countries and pathogen targets, including SARS-CoV-2 in the United States [43], Mpox in Spain [44], *S. typhi* in Malawi [45], and multi-pathogen surveillance in India [4].

In each example case study, we describe the choices made by leading wastewater surveillance teams, and how these compare to, and could be strengthened through guidance document recommendations. For instance, we discuss the trade-offs for grab versus passive sampling approaches when sampling from rivers and surface waters, and for the use of time-weighted and flow-weighted composite sampling approaches where infrastructure permits. This informal, practical approach lowers the barrier to entry for those looking to initiate wastewater surveillance programs, and highlights best practice implementations and potential areas for improvement. As wastewater surveillance technology continues to evolve, these examples will be periodically revised to reflect priority pathogens and public health objectives.

To further operationalize the application of key legal, ethics, equity, and governance principles in everyday surveillance operations, the PHA4GE Ethics and Data Sharing working group, together with members of the African Data and Biospecimen Exchange and the Pathogen Data Network have developed an interactive online tool, the Wastewater Ethics Advisor App [46]. The app provides a user-friendly walkthrough with alerts for practitioners on important factors and considerations for WES implementation and data sharing. Users can select their specific sample type and origin, and the app returns tailored guidance including on potential benefits and harms, steps to support equitable deployment, and recommended governance, data protection, and data access practices.

## Community-led development and sustainability

To maintain public health community standards that reflect technological capabilities and global public health priorities, we have designed these best-practice guidelines as “living” resources that can be openly accessed and updated over time. Although most suggestions to date have come directly from group members, the framework is intentionally community-driven: anyone can provide feedback or direct updates through Github with open access and transparent version tracking. These proposed updates are then reviewed and discussed during biweekly group meetings and incorporated into the guidance documents once consensus is reached.

For many scientists looking to get started with wastewater surveillance, guidance documents alone are insufficient, and basic training on core methods and concepts is needed [47]. To provide essential background and skill development, we have developed comprehensive tutorial materials covering topics from sample collection to bioinformatics and data sharing. These training modules and in-person workshops contain lessons grounded in realistic public health challenges, providing researchers with a user-friendly entry point into complex fields such as bioinformatics [48]. We plan to maintain these as open-access resources and are pursuing accreditation to support learners who may benefit from formal certification.

Sustaining this effort requires long-term commitment from public health scientists and stakeholders. Just as the wastewater guidance itself has been developed without any dedicated funding, we will continue to largely rely on the efforts of volunteers, especially from PHA4GE, as well as other scientists utilizing these resources and providing feedback. However, we recognize that volunteer efforts are dependent on member availability, are generally insufficient for sustained resource development, and limit the involvement of group members in low- and middle-income settings. For activities requiring dedicated and consistent effort, we have begun implementing targeted contracts, including for development of WES training materials and evaluation of guideline usage in public health settings. PHA4GE has also supported members to present at conferences and lead WES workshops, expanding public health community WES expertise and supporting continued professional development.

## Conclusion

Over the last decade, we have seen the emergence of a novel coronavirus that caused a global pandemic, a human-adapted strain of Mpox, and spillover of avian influenza into a new mammalian host, all of which relied on WES to answer important public health questions and guide outbreak responses. By harnessing the potential of WES, we have an opportunity to revolutionize infectious disease surveillance, especially for vulnerable and underserved groups, and move towards a future where laboratories worldwide both adhere to and contribute to best practices. Community-led standards that embed ethics, equity, and openness are essential for trustworthy and globally inclusive public health surveillance, and adaptability and scalability ensure they can be used wherever and whenever the next pathogen with pandemic potential emerges.
